# The gene expression patterns of BMPR2, EP300, TGFβ2, and TNFAIP3 in B-Lymphoma cells

**DOI:** 10.7497/j.issn.2095-3941.2014.03.006

**Published:** 2014-09

**Authors:** Dong-Mei He, Hong Wu, Xiu-Li Wu, Li Ding, Ling Xu, Yang-Qiu Li

**Affiliations:** ^1^Institute of Hematology, Medical College, ^2^Key Laboratory for Regenerative Medicine of Ministry of Education, Jinan University, Guangzhou 510632, China

**Keywords:** Bone morphogenetic protein receptor, type II (*BMPR2*), E1A binding protein p300 (*EP300*), transforming growth factor-β2 (*TGFβ2*), tumor necrosis factor, and alpha-induced protein 3 (*TNFAIP3*), B-lymphoma cells, myeloid leukemia cells, quantitative reverse transcription polymerase chain reaction (qRT-PCR)

## Abstract

**Objective:**

The results of a previous study showed that a clear dysregulation was evident in the global gene expression of the BCL11A-suppressed B-lymphoma cells. In this study, the bone morphogenetic protein receptor, type II (*BMPR2*), E1A binding protein p300 (*EP300*), transforming growth factor-β2 (*TGFβ2*), and tumor necrosis factor, and alpha-induced protein 3 (*TNFAIP3*) gene expression patterns in B-cell malignancies were studied.

**Methods:**

The relative expression levels of *BMPR2*, *EP300*, *TGFβ2*, and *TNFAIP3* mRNA in B-lymphoma cell lines, myeloid cell lines, as well as in cells from healthy volunteers, were determined by real-time quantitative reverse transcript-polymerase chain reaction (qRT-PCR) with SYBR Green Dye. Glyceraldehyde-3-phosphate dehydrogenase (*GAPDH*) was used as reference.

**Results:**

The expression level of *TGFβ2* mRNA in B-lymphoma cell lines was significantly higher than those in the cells from the healthy control (*P*<0.05). However, the expression level of *TNFAIP3* mRNA in B-malignant cells was significantly lower than that of the healthy control (*P*<0.05). The expression levels of *BMPR2* and *EP300* mRNA showed no significant difference between B-malignant cell lines and the healthy group (*P*>0.05). In B-lymphoma cell lines, correlation analyses revealed that the expression of *BMPR2* and *TNFAIP3* (*r*=0.882, *P*=0.04) had significant positive relation. The expression levels of *BMPR2*, *EP300*, and *TNFAIP3* mRNA in cell lines from myeloid leukemia were significantly lower than those in the cells from the healthy control (*P*<0.05). The expression levels of *TGFβ2* mRNA showed no significant difference between myeloid leukemia cell lines and the healthy control or B-malignant cell lines (*P*>0.05). The expression levels of *BMPR2*, *EP300*, and *TNFAIP3* mRNA in B-lymphoma cells were significantly higher than those of the myeloid leukemia cells (*P*<0.05).

**Conclusion:**

Different expression patterns of *BMPR2*, *EP300*, *TGFβ2*, and *TNFAIP3* genes in B-lymphoma cells exist.

## Introduction

Non-Hodgkin lymphomas (NHLs) are the fifth most frequently occurring cancer worldwide^1^^-^^3^. Diffuse large B-cell lymphoma (DLBCL) is considered a highly heterogeneous type of NHL in its morphology, immunophenotypical, biological, and clinical aspects. Gene expression profile analysis has identified a germinal-center (GC) B-cell-like, an activated B-cell-like (ABC), and an intermediate type of DLBCL with different prognostic implications^4^^,^^5^. Burkitt’s lymphoma (BL) is an aggressive B-cell NHL, characterized by a high degree of proliferation of the malignant cells. The main diagnostic challenge in BL is to distinguish it from DLBCL^6^^,^^7^.

B-cell chronic lymphocytic leukemia (CLL)/lymphoma 11 (*BCL-11A*) gene is associated with human malignant B cells, overexpression of which primarily occurs in B-cell lymphoma and B-cell leukemia^8^^-^^11^. A previous study has shown that a broad range of genes may be altered in B-cell lymphoma, including bone morphogenetic protein receptor, type II (*BMPR2*), E1A binding protein p300 (*EP300*), transforming growth factor-β (*TGFβ2*), tumor necrosis factor, and alpha-induced protein 3 (*TNFAIP3*) gene (also known as *A20*), by analyzing the global gene expression profile in the GCB-derived DLBCL cell line SUDHL6 after *BCL11A* downregulation (in press).

Bone morphogenetic proteins (BMPs) are members of TGF-β superfamily of signaling molecules. Deregulation of BMPs signaling pathways has been reported in certain human cancers. *BMPR2* has tumor-suppressive functions in mammary carcinoma and B-CLL^12^^,^^13^. *BMPR2* was strongly expressed in acute promyelocytic leukemia, as well as in AML-M4 and multiple myeloma^14^. However, the role of BMPs in B-lymphoma remains unknown. The *EP300* gene encodes the adenovirus E1A-associated cellular p300 transcriptional co-activator protein. It is important in the processes of cell proliferation and differentiation^15^^,^^16^. *TNFAIP3* is a putative tumor suppressor gene in B-cell lymphomagenesis and the frequency of *TNFAIP3* gene inactivation was observed in different subtypes of NHL^17^.

Although *BMPR2*, *EP300*, *TGFβ2*, and *TNFAIPI3* have been studied in lymphoma or leukemia, no report has compared the expression of the four genes among B-lymphoma cells, the healthy control, and myeloid leukemia. In this study, quantitative reverse transcription polymerase chain reaction (qRT-PCR) was used to analyze the expression levels of *BMPR2*, *EP300*, *TGFβ2*, and *TNFAIPI3* in B-lymphoma and myeloid leukemia cell lines.

## Materials and methods

### Cell lines

Human B-lymphoma cell lines (DG-75, EB1, OCI-LY-3, and SUDHL6) were kindly provided by Prof. Ailin Guo (Institute of Pathology, Cornell University). Human B-lymphoma cell lines (Daudi, Raji) and myeloid leukemia cell lines (HL-60, NB4, U937, and K562) were purchased from a cell bank in Shanghai, China. The cells were cultured at 37 °C in an atmosphere containing 5% CO_2_ in RPMI-1640 medium supplemented with penicillin (100 U/mL), streptomycin (0.1 mg/mL), and 10% fetal calf serum.

Ten healthy volunteers served as control (seven males and three females, 22-62 years old; median age, 31 years). All procedures were conducted in accordance with the guidelines of Medical Ethics committees of the health bureau of Guangdong Province, PR China. Peripheral blood was collected by heparin anticoagulation. Peripheral blood mononuclear cells (PBMNCs) were separated with the use of the Ficoll-Hypaque gradient centrifugation method.

### RNA extraction and cDNA synthesis

RNA was extracted using the Trizol kit (Invitrogen, Carlsbad, CA, USA) and reversely transcribed into the first-strand cDNA with the use of random hexamer primers and the reverse transcriptase Superscript II Kit (Invitrogen, USA), according to the manufacturer’s instructions. RNA purity and concentration was measured with a spectrophotometer. The integrity of RNA was checked on a 1% agarose gel. Glyceraldehyde-3-phosphate dehydrogenase (*GAPDH*) gene by qRT-PCR was used to determine the quality of the synthesized cDNA.

### Real-time quantitative PCR with SYBR green I

The 2^–ΔCt^ ×100% method could be used to analyze the relative changes in gene expression from real-time quantitative PCR experiments^18^^,^^19^. Before applying the method to detect gene expression level, the targeted genes and the *GAPDH* gene should be determined to have high amplification efficiency. Therefore, a validation experiment was conducted using template serial dilutions of the cell lines cDNA covering the five orders of magnitude, 0.0001, 0.001, 0.01, 0.1, and 1. Standard curves were generated by plotting the Ct values against the logarithm starting quantity of the series dilution of cDNA template. The slope and correlation coefficient (R2) were determined by linear regression analysis. Briefly, the primers were designed and synthesized (Invitrogen, USA). The following primer sequences used in PCR reactions were shown in **Table 1**. The total reaction volume is 20 μL. Reaction conditions started with enzyme activation at 95 °C for 10 min, followed by 40 cycles of 95 °C for 15 s, 60 °C for 30 s, and 80 °C for 5 s. At the end of each run, a melting curve was performed starting at 65 °C to 95 °C with an increase of 1 °C per 2 s to verify primer specificities. Each run was completed with a melting curve analysis to confirm the specificity of the amplification and the absence of primer dimmers. The qRT-PCR was repeated in at least three separate experiments. The PCR products were visualized and separated on 2% agarose gel electrophoresis.

**Table 1:** Primer sequences for real-time polymerase chain reaction

Primer	Sequence	Size (bp)
BMPR2-f	5’ GGCTGAACTTATGATGATTTGGGAA 3’	107
BMPR2-r	5’ CACGCCTATTATGTGACAGGTTGC 3’	
EP300-f	5’ TCCGAGACATCTTGAGACGACAG 3’	107
EP300-r	5’ GGGTTGCTGGAACTGGTTATGG 3’	
TGFβ2-f	5’ GTTCGATTTGACGTCTCAGCAAT 3’	107
TGFβ2-r	5’ CAATCCGTTGTTCAGGCACTCT	
TNFAIP3-f	5’ CCACAAAGCCCTCATCGACAG 3’	109
TNFAIP3-r	5’ GTCACCGTTCGTTTTCAGCG 3’	
GAPDH-f	5’ ACCCAGAAG ACTGTGGATGG 3’	114
GAPDH-r	5’ TTCAGCTCA GGG ATGACCTT 3’	

### Statistical analysis

Nonparametric test analysis of two independent samples was used for the relative expression levels of gene mRNA in different samples, whereas the Mann-Whitney U test was used for non-normally distributed data using the SPSS 13.0 statistical software. Differences were considered statistically significant at *P*<0.05.

## Results

### PCR product analysis

R2 values of standard curves for *BMPR2*, *EP300*, *TGFβ2*, and *TNFAIP3* reactions are above 0.99. The amplification efficiencies of the four genes and the *GAPDH* control gene were above 95%. The high amplification efficiency of the four genes was consistent with that of the *GAPDH* reference gene. The PCR products from the *GAPDH* control gene and the four genes were confirmed using 2% gel electrophoresis (data not shown).

### The expression levels of *BMPR2*, *EP300*, *TGFβ2*, and *TNFAIP3* genes in B-lymphoma cell lines

**Figure 1:**
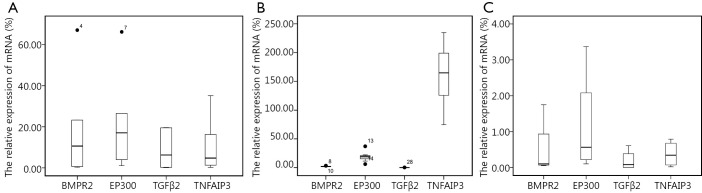
The relative expression levels of *BMPR2*, *EP300*, *TGFβ2*, and *TNFAIP3* mRNA in B-lymphoma cell lines and myeloid leukemia cell lines. Results shown are the boxplots of the expression levels of the genes. Numbers 4, 7 (A) and 8, 10, 13, 14, 28 (B) represent singular value. The expression levels of *TGFβ2* and *TNFAIP3* mRNA were found to be significantly different between the B-lymphoma cell lines (A) and the healthy control (B) (*P*<0.05). The expression levels of *BMPR2*, *EP300*, and *TNFAIP3* mRNA were revealed to be significantly different between the myeloid leukemia cell lines (C) and the healthy control (*P*<0.05).

Ten malignancy hematology cell lines (six B-malignancy cells and four myeloid cells) were assayed for RNA expression levels. B-malignancy cells include Burkitt’s lymphoma cell lines (EB1, Daudi, DG75, and Raji) and GCB DLBCL-derived cell lines (OCI-LY-3 and SUDHL6). The mRNA expression level for each of the four genes was normalized to the reference gene (*GAPDH*). The ∆Ct was calculated for all four genes. According to the relative qRT-PCR formula: 2^–∆Ct^ ×100%, the relative expression level of *TGFβ2* mRNA in cell lines from B-cell malignancies was significantly higher than that in the cells from the healthy control (*P*<0.05). On the other hand, the expression level of *TNFAIP3* mRNA in B-malignant cells was significantly lower than that of the healthy control (*P*<0.05). As shown in **Figure 1**, the median quantities of *TGFβ2* and *TNFAIP3* expression in these B-cell lines are 6.25% and 4.78%, respectively, whereas the relative median expression levels of the two genes in the healthy group are 0.12% and 164.74%. The relative expression levels of *BMPR2* and *EP300* mRNA indicated no significant difference between B-cell malignant cell lines and PBMNCs of the healthy group (*P*>0.05).

In B-lymphoma cell lines, correlation analyses revealed that the expression of *BMPR2* and *TNFAIP3* (*r*=0.882, *P*=0.04) had significant positive relation.

### The expression levels of *BMPR2*, *EP300*, *TGFβ2*, and *TNFAIP3* genes in myeloid leukemia cells

The relative expression levels of *BMPR2*, *EP300*, and *TNFAIP3* mRNA in cell lines from myeloid leukemia were significantly lower than those in the cells from the healthy people (*P*<0.05). As shown in **Figure 1**, the median quantities of *BMPR2*, *EP300*, and *TNFAIP3* expression in the myeloid leukemia cell lines are 0.10%, 0.56%, and 0.34%, respectively. Furthermore, the relative median expression levels of these genes in the healthy control are 2.04%, 19.05%, and 164.74%, respectively. The relative expression levels of *TGFβ2* mRNA showed no significant difference between myeloid leukemia cell lines and the healthy control (*P*>0.05).

### *BMPR2*, *EP300*, *TGFβ2*, and *TNFAIP3* gene expression in B-malignant and myeloid leukemia cell lines

As shown in **Figure 1**, the relative expression levels of *BMPR2*, *EP300*, and *TNFAIP3* mRNA in the cell lines from B-cell malignancy were significantly higher than those cell lines from myeloid leukemia (*P*<0.05), whereas the relative expression levels of *TGFβ2* mRNA showed no significant difference between B-cell malignant cell lines and myeloid leukemia cell lines (*P*>0.05).

## Discussion

In this study, *BMPR2*, *EP300*, *TGFβ2*, and *TNFAIP3* mRNA expression were determined by qRT-PCR using SYBR Green I dye. Results showed that the high amplification efficiency of these genes was consistent with that of *GAPDH*, suggesting that the 2^–∆Ct^ ×100% method can be used in quantifying the relative expression levels of the gene mRNA. This study revealed the expression of *TGFβ2* and *TNFAIP3* mRNA in B-malignant cells were significantly different from those of the healthy control. Meanwhile, the expression levels of *BMPR2*, *EP300*, and *TNFAIP3* mRNA in myeloid leukemia cells were significantly lower than those of healthy people.

Deregulated or aberrant TGF-β signaling has been strongly implicated in the pathogenesis of human solid tumors, while less is known about the role of this pathway in lymphoma pathogenesis^20^. TGF-β has either a tumor-suppressing or tumor-promoting function depending on cellular context^21^. Moreover, TGF-β is overexpressed in chronic lymphocytic leukemia. TGF-β functions as an autocrine growth inhibitor in chronic lymphocytic leukemia B-cells^22^. In the study of Ho *et al*.^23^, *TGFβ2* was expressed in normal B-cell differentiation of the germinal center, especially from the follicular center cell stage to the postfollicular plasma cell stage. However, there was no expression of TGF-β in specimens from patients with neoplastic NHL. In this study, the expression level of *TGFβ2* mRNA in PBMNCs from the healthy control is almost lacking. The expression level of *TGFβ2* mRNA in the cell lines from B-malignancies was significantly higher than those in the cells from the healthy control. Furthermore, TGF-β functions as either suppressing or promoting the effect on tumors need to be investigated. *TNFAIP3* inactivation has been reported in various B-cell lymphomas. In GCB DLBCL, the expression of *TNFAIP3* was lower than that in ABC DLBCL^17^. Meanwhile, Paik *et al*.^24^ observed that *TNFAIP3* deletion was in similar frequencies in GCB and non-GCB/ABC DLBCL using fluorescence *in situ* hybridization. These contradictory observations may reflect the diversified roles of *TNFAIP3* in DLBCL. These results showed that the expression level of *TNFAIP3* mRNA in B-malignant cells was significantly lower than that of the healthy control, suggesting possible deletion of *TNFAIP3* gene in the B-lymphoma cells, which is in accordance with previous observations^23^. More so, *TNFAIP3* gene was expressed at lower levels in GCB DLBCL cell lines than in Burkitt’s lymphoma cell lines.

*EP300* mutations were recently identified as a major pathogenetic mechanism shared by common forms of B-cell NHLs^25^. By contrast, the findings in this study demonstrate that the expression levels of *BMPR2* and *EP300* mRNA had no significant difference between B-cell malignant cell lines and PBMNCs of the healthy people.

Results indicate that the expression levels of *BMPR2*, *EP300*, and *TNFAIP3* mRNA in myeloid leukemia cell lines were significantly lower than those of healthy people or B-malignant cell lines. *BMPR2*, *EP300*, and *TNFAIP3* mRNA have been observed to be almost absent in myeloid leukemia cell lines. This observation indicates that the expression patterns of *BMPR2*, *EP300*, and *TNFAIP3* in B-cell lymphoma may be significantly different from myeloid leukemia cell lines. Although the median quantity of *TGFβ2* expression in B-lymphoma cell lines is obviously higher than that of the myeloid leukemia cell lines, no statistically significant difference is observed. This finding might denote a large variance of *TGFβ2* expression level (three orders of magnitude) in the B-lymphoma cell lines. Additionally, in the different B-lymphocyte malignant cell lines, the expression levels of *BMPR2*, *EP300*, *TGFβ2*, and *TNFAIP3* mRNA were quite different, which may be related to the heterogeneity of B-cell lymphoma.

Little is known about the expression correlation between *BMPR2* and *TNFAIP3* genes in B-cell lymphoma. This study showed that the expression of *BMPR2* and *TNFAIP3* had a significant positive relation in B-lymphoma cell lines. These results indicate that a positively correlated expression pattern may be a feature in B-cell lymphoma. However, increasing the number of samples for validating the expression relation of the two genes is needed.

In conclusion, the expression patterns of *BMPR2*, *EP300*, *TGFβ2*, and *TNFAIP3* genes were characterized in B-lymphoma cells. Meanwhile, the differential expression pattern needs to increase the sample numbers to be further studied in B-cell malignancies. In addition, further biological studies are needed to determine if *TGFβ2* and *TNFAIP3* are therapeutic targets for B-cell lymphoma.

## References

[1] Deffenbacher KE Iqbal J Sanger W Shen Y Lachel C Liu Z Molecular distinctions between pediatric and adult mature B-cell non-Hodgkin lymphomas identified through genomic profiling. Blood 2012;119:3757-3766 22374697 10.1182/blood-2011-05-349662 PMC3335381

[2] Jemal A Siegel R Ward E Murray T Xu J Thun MJ Cancer statistics, 2007. CA Cancer J Clin 2007;57:43-66 17237035 10.3322/canjclin.57.1.43

[3] Gloeckler Ries LA Reichman ME Lewis DR Hankey BF Edwards BK Cancer survival and incidence from the Surveillance, Epidemiology, and End Results (SEER) program. Oncologist 2003;8:541-552 14657533 10.1634/theoncologist.8-6-541

[4] Alizadeh AA Eisen MB Davis RE Ma C Lossos IS Rosenwald A Distinct types of diffuse large B-cell lymphoma identified by gene expression profiling. Nature 2000;403:503-511 10676951 10.1038/35000501

[5] Hans CP Weisenburger DD Greiner TC Gascoyne RD Delabie J Ott G Confirmation of the molecular classification of diffuse large B-cell lymphoma by immunohistochemistry using a tissue microarray. Blood 2004;103:275-282 14504078 10.1182/blood-2003-05-1545

[6] Bellan C Stefano L, Giulia de F, Rogena EA, Lorenzo L. Burkitt lymphoma versus diffuse large B-cell lymphoma: a practical approach. Hematol Oncol 2010;28:53-56 19844983 10.1002/hon.916

[7] Dave SS Fu K Wright GW Lam LT Kluin P Boerma EJ Molecular diagnosis of Burkitt’s lymphoma. N Engl J Med 2006;354:2431-2442 16760443 10.1056/NEJMoa055759

[8] Satterwhite E Sonoki T Willis TG Harder L Nowak R Arriola EL The BCL11 gene family: involvement of BCL11A in lymphoid malignancies. Blood 2001;98:3413-3420 11719382 10.1182/blood.v98.12.3413

[9] Liu H Ippolito GC Wall JK Niu T Probst L Lee BS Functional studies of BCL11A: characterization of the conserved BCL11A-XL splice variant and its interaction with BCL6 in nuclear paraspeckles of germinal center B cells. Mol Cancer 2006;5:18 16704730 10.1186/1476-4598-5-18 PMC1526750

[10] Yin B Delwel R Valk PJ Wallace MR Loh ML Shannon KM A retroviral mutagenesis screen reveals strong cooperation between Bcl11a overexpression and loss of the Nf1 tumor suppressor gene. Blood 2009;113:1075-1085 18948576 10.1182/blood-2008-03-144436 PMC2635073

[11] Gao Y He D Chen S Yan X Hu X Li Y. Expression of the B-cell lymphoma/leukemia 11A gene in malignant hematological cell lines through quantitative reverse transcription polymerase chain reaction. Clin Oncol Cancer Res 2011;8:242-246

[12] Dzietczenia J Wróbel T Jaźwiec B Mazur G Butrym A Poręba R Expression of bone morphogenetic proteins (BMPs) receptors in patients with B-cell chronic lymphocytic leukemia (B-CLL). Int J Lab Hematol 2010;32:e217-221 20491995 10.1111/j.1751-553X.2010.01233.x

[13] Owens P Pickup MW Novitskiy SV Chytil A Gorska AE Aakre ME Disruption of bone morphogenetic protein receptor 2 (BMPR2) in mammary tumors promotes metastases through cell autonomous and paracrine mediators. Proc Natl Acad Sci U S A 2012;109:2814-2819 21576484 10.1073/pnas.1101139108 PMC3286911

[14] Grcević D Marusić A Grahovac B Jaksić B Kusec R. Expression of bone morphogenetic proteins in acute promyelocytic leukemia before and after combined all trans-retinoic acid and cytotoxic treatment. Leuk Res 2003;27:731-738 12801531 10.1016/s0145-2126(02)00281-3

[15] Garbati MR Alço G Gilmore TD Histone acetyltransferase p300 is a coactivator for transcription factor REL and is C-terminally truncated in the human diffuse large B-cell lymphoma cell line RC-K8. Cancer Lett 2010;291:237-245 19948376 10.1016/j.canlet.2009.10.018 PMC2849871

[16] Iyer NG Ozdag H Caldas C p300/CBP and cancer. Oncogene 2004;23:4225-4231 15156177 10.1038/sj.onc.1207118

[17] Honma K Tsuzuki S Nakagawa M Tagawa H Nakamura S Morishima Y TNFAIP3/A20 functions as a novel tumor suppressor gene in several subtypes of non-Hodgkin lymphomas. Blood 2009;114:2467-2475 19608751 10.1182/blood-2008-12-194852

[18] Livak KJ Schmittgen TD Analysis of relative gene expression data using real-time quantitative PCR and the 2(-Delta Delta C(T)) Method. Methods 2001;25:402-408 11846609 10.1006/meth.2001.1262

[19] Papakonstantinou G Verbeke C Hastka J Bohrer M Hehlmann R. bcl-2 expression in non-Hodgkin’s lymphomas is not associated with bcl-2 gene rearrangements. Br J Haematol 2001;113:383-390 11380403 10.1046/j.1365-2141.2001.02727.x

[20] Lin HK Bergmann S Pandolfi PP Deregulated TGF-beta signaling in leukemogenesis. Oncogene 2005;24:5693-5700 16123802 10.1038/sj.onc.1208923

[21] Ikushima H Miyazono K. TGFbeta signalling: a complex web in cancer progression. Nat Rev Cancer 2010;10:415-424 20495575 10.1038/nrc2853

[22] Lotz M Ranheim E Kipps TJ Transforming growth factor beta as endogenous growth inhibitor of chronic lymphocytic leukemia B cells. J Exp Med 1994;179:999-1004 8113691 10.1084/jem.179.3.999 PMC2191408

[23] Ho CL Sheu LF Li CY Immunohistochemical expression of angiogenic cytokines and their receptors in reactive benign lymph nodes and non-Hodgkin lymphoma. Ann Diagn Pathol 2003;7:1-8 12616467 10.1053/adpa.2003.50000

[24] Paik JH Go H Nam SJ Kim TM Heo DS Kim CW Clinicopathologic implication of A20/TNFAIP3 deletion in diffuse large B-cell lymphoma: an analysis according to immunohistochemical subgroups and rituximab treatment. Leuk Lymphoma 2013;54:1934-1941 23327292 10.3109/10428194.2012.762511

[25] Pasqualucci L Dominguez-Sola D Chiarenza A Fabbri G Grunn A Trifonov V Inactivating mutations of acetyltransferase genes in B-cell lymphoma. Nature 2011;471:189-195 21390126 10.1038/nature09730 PMC3271441

